# An Opto‐Bio‐Hydrodynamic Platform for Instructing Cardiac Left‐Right Asymmetry Development

**DOI:** 10.1002/advs.202512368

**Published:** 2025-08-18

**Authors:** Haifeng Qin, Xiaoshuai Liu, Yufeng Lin, Guangyi Yang, Haojiang Ren, Mingyuan Cao, Zhenheng Jiao, Baojun Li, Xianchuang Zheng

**Affiliations:** ^1^ Guangdong Provincial Key Laboratory of Nanophotonic Manipulation Institute of Nanophotonics College of Physics & Optoelectronic Engineering Jinan University Guangzhou 511443 China; ^2^ School of Physics and Materials Science Guangzhou University Guangzhou 510006 China

**Keywords:** biophotonics, cardiac development, ciliary motion, left‐right asymmetry, optical manipulation

## Abstract

The spatiotemporal regulation of ciliary dynamics within the left‐right organizer (LRO) governs cardiac laterality establishment through biomechanical signaling gradients. While targeted restoration of aberrant ciliary motion can theoretically rescue left‐right patterning defects, existing manipulation strategies lack the non‐invasiveness, micron‐scale precision, and spatiotemporal programmability required for developmental interventions. Here, a multifunctional Opto‐Bio‐Hydrodynamic platform is presented to modulate ciliary motion with the programmable near‐infrared light for instructing cardiac left‐right asymmetry development in a spatiotemporally controlled manner. By the real‐time modulation of a 1064‐nm light beam, a direct optical trapping is demonstrated for the motile cilia, while uniform synchronization of ciliary beating is achieved through indirect optical regulation. Consequently, a coordinated ciliary rotational pattern triggers an enhanced recirculating flow, under which the methylcellulose‐induced cardiogenesis abnormality is rescued in a fully controlled manner. By establishing mechanistic links among optically tuned ciliary dynamics, flow‐mediated signaling, and organ asymmetry, this proposed strategy provides valuable insights into congenital heart defects and offers a promising biomedical platform for developmental bioengineering and mechanobiological therapeutics.

## Graphical abstract

By establishing mechanistic links among optically tuned ciliary dynamics, flow‐mediated signaling, and organ asymmetry, a multifunctional Opto‐Bio‐Hydrodynamic platform is developed to modulate ciliary motion with the programmable near infrared light for instructing cardiac left‐right asymmetry development in a spatiotemporally controlled manner, which provides an innovative tool to uncover universal principles underlying organ asymmetry development and enables an early intervention in congenital heart diseases, visceral heterotaxy, and related disorders.

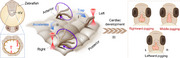

## 1 Introduction

In healthy humans, visceral organs exhibit a conserved left‐right asymmetric spatial pattern,^[^ ^1^, ^2^, ^3^, ^4^ ^]^ i.e., the heart is positioned on the left thoracic cavity rather than right, which facilitates both functional specialization, such as systemic‐pulmonary circulatory separation, and spatial optimization of intrathoracic organs. However, ≈0.01% of the population manifests the situs inversus with a complete mirror‐image organ reversal where the heart resides in the right hemithorax.^[^ ^5^, ^6^, ^7^, ^8^ ^]^ Crucially, such cases usually present with concurrent congenital cardiac defects and multiorgan malformations,^[^ ^9^, ^10^ ^]^ exhibiting high mortality rates within the first postnatal year.^[^ ^11^, ^12^ ^]^ Therefore, considerable research interests have been focused on elucidating the spatiotemporal regulation of cardiac laterality establishment, with the aim of enabling early intervention in aberrant developmental processes and potentially guiding cardiogenesis toward typical left‐sided morphogenesis.^[^ ^4^, ^13^, ^14^ ^]^

Notably, the spatial pattern of organ distribution is critically dependent on the transient left‐right organizer (LRO) during embryogenesis, in which endogenous cilia generate directional extracellular fluid flow through coordinated beating patterns, thereby establishing biomechanical signaling gradients by the dynamic enrichment of signaling proteins.^[^ ^15^, ^16^, ^17^, ^18^, ^19^ ^]^ Based on continuous beating, motile cilia drive leftward fluid flow to facilitate asymmetric accumulation of signaling molecules on the left, followed by the dynamic activation of downstream gene cascades.^[^ ^20^, ^21^, ^22^, ^23^ ^]^ Concurrently, immotile cilia can transduce fluid shear stress via the Pkd2 channel, as well as convert mechanical cues into biochemical instructions that determine the specific positioning of organ primordia.^[^ ^18^, ^24^, ^25^, ^26^ ^]^ Inspired by this mechanism, targeted restoration of dysregulated ciliary motility through spatiotemporally precise modulation can mitigate aberrant cardiogenesis, thereby reorganizing disordered left‐right axis patterning and reducing the incidence of congenital anomalies such as cardiac heterotaxia and visceral situs inversus.^[^ ^14^, ^27^, ^28^, ^29^ ^]^

To realize this purpose, innovative intervention modalities have been developed for spatiotemporal control of ciliary activity, including the pharmacological modulation via small molecules,^[^ ^16^ ^]^ biomechanical stimulation,^[^ ^30^ ^]^ acoustic tweezer manipulation,^[^ ^31^ ^]^ and magnetically actuated systems.^[^ ^32^ ^]^ Although enabling effective control of ciliary dynamics, existing approaches lack the combined advantages of noninvasive operation, micrometer‐scale precision, and flexible programmability required for developmental bioengineering. Optical tweezers, emerging as a fascinating micromanipulation strategy, have revolutionized the research paradigm ranging from single cell,^[^ ^33^, ^34^, ^35^, ^36^ ^]^ subcellular architecture,^[^ ^37^, ^38^, ^39^ ^]^ even to biomacromolecules,^[^ ^40^, ^41^, ^42^ ^]^ which should be ascribed to their contactless ability, micron positioning resolution, and programmable flexibility. In this study, we demonstrate a programmable Opto‐Bio‐Hydrodynamic platform to conduct the precise modulation of cardiac left‐right asymmetric development by manipulating ciliary motion with programmable near‐infrared light. Under the action of optical gradient force, the target cilia can experience a stable trapping by a tightly focused laser beam. Meanwhile, the introduced laser beam enables systemic modulation across all cilia within LRO, which can be explained by the photothermal activation of calcium channels in hair cells with the intercellular signaling cascades. Consequently, a coordinated rotational pattern will trigger an enhanced recirculating flow, under which the abnormal left‐right asymmetric development can be rescued in a fully controlled manner. This reported technique not only holds promise to uncover universal principles underlying organ asymmetry development, but also provides a theoretical basis and research strategy for early intervention in congenital heart diseases, visceral heterotaxy, and related disorders.

## 2 Results

### 2.1 Schematic Illustration and Material Characterization

**Figure 1:**
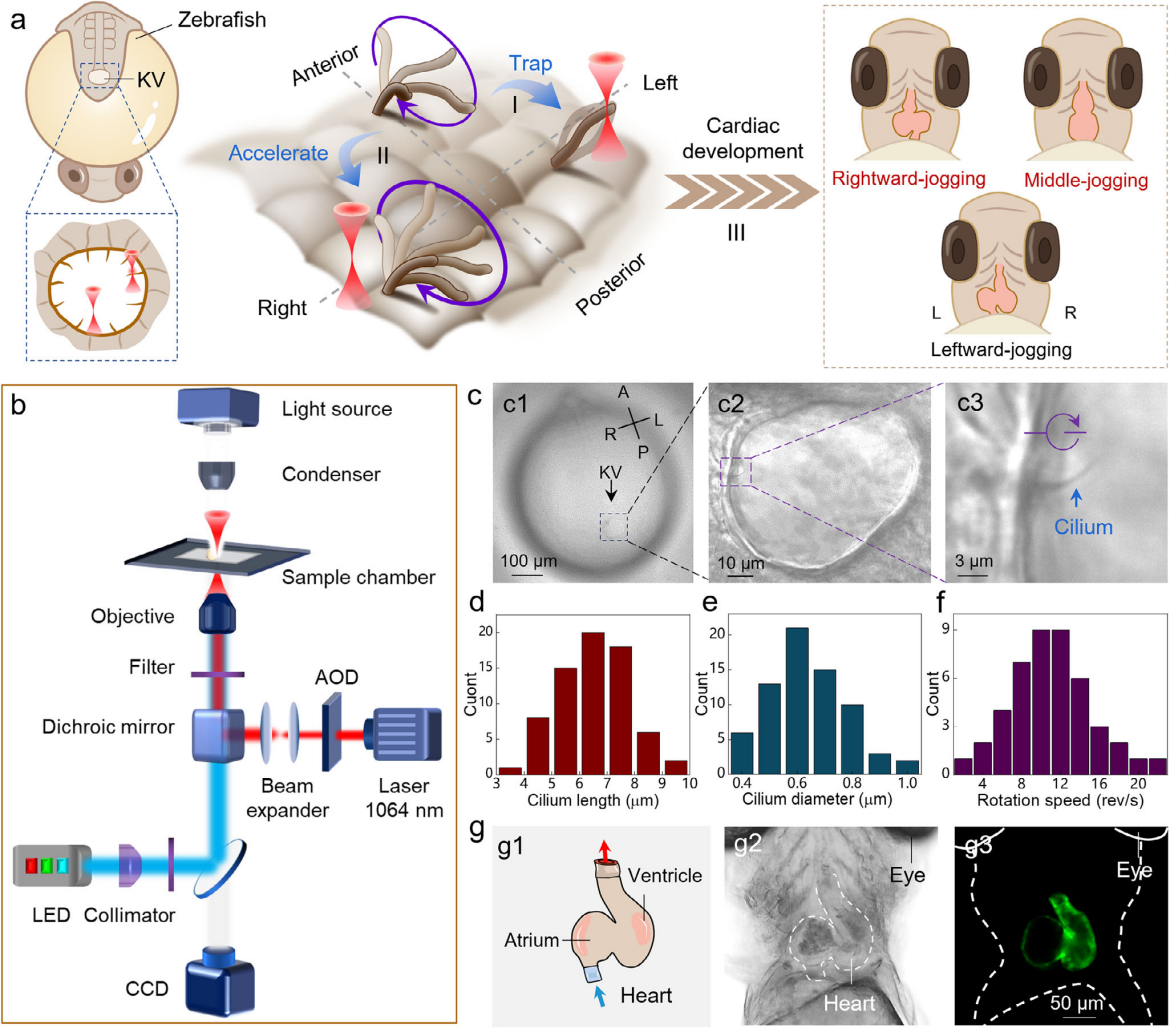
Schematic illustration and material characterization. a) Design rationale of instructing left‐right asymmetry development through cilia modulation in vivo. b) Schematic illustration of experimental setup. c) Optical microscopic images of zebrafish embryo (c1), LRO (c2), and cilium (c3). d–f) Quantitative analysis of the length (d), diameter (e), and rotational speed (f) of cilia within LRO. g) Schematic diagram (g2), bright‐field (g2) and fluorescence image (g3) of zebrafish heart (5 dpf).

**Figure** **1**a indicates the schematic illustration of the Opto‐Bio‐Hydrodynamic platform for instructing left‐right asymmetry development by manipulating the ciliary motion with near‐infrared light. The cilia are located in the Kupffer's vesicle (KV), also known as LRO, and grow above the hair cells with an autonomous rotation, i.e., clockwise or anticlockwise. Under the actuation of cilia rotation, the biofluid within LRO starts to flow, whose velocity and direction are strongly dependent on the specific rotation modes of cilia. On this basis, a desired microflow field can be expected by modulating the cilia motion with a light beam, which can be divided into two strategies, including the direct optical trapping and indirect optical regulation. For the direct optical trapping, when the light beam is directed to irradiate on the distal tip of target cilium (Figure 1aI), momentum transfer via light‐matter interactions generates optical forces comprising two components: optical scattering force (*F*_s_) and optical gradient force (*F*_g_), whose direction was aligned with laser propagation and directed toward the beam focus, respectively. Concurrently, the ciliary tip will move toward the optical axis under the action of *F*_g_, after which *F*_g_ will compete with *F*_s_, and a stable trapping could be achieved with the force balance in favor of *F*_g_. On the other hand, the light beam can also be focused on the non‐ciliary position while being directly exerted on the hair cells. Consequently, the absorption‐induced heat enables a powerful simulation on the membrane potential, which will spread quickly across the surrounding hair cells due to the tight intercellular connections,^[^ ^43^, ^44^ ^]^ leading to a synchronous modulation of the cilia within LRO. This indirect optical regulation can sculpt the microflow field in a real‐time and programmable manner (Figure 1aII). Subsequently, the sculpted microflow will trigger a significant increase in calcium activity and modulate the expression of asymmetric genes,^[^ ^16^, ^17^ ^]^ thereby enabling a successful instruction of left‐right asymmetry for cardiac development, such as leftward‐jogging, middle‐jogging, and rightward‐jogging phenotypes (Figure 1aIII). To enable the multifunctional manipulation and long‐term characterization of cilia motion, a scanning optical tweezer system (SOTs) was introduced, which was constructed around the inverted fluorescence microscope (Figure 1b). The incident laser beam, at a wavelength of 1064 nm, interacted with the acoustic‐optic deflector (AOD) to achieve spatiotemporal modulation of beam focus while with a maximum switching rate of 100 KHz.

In this study, the zebrafish were chosen as the animal model due to their excellent optical transparency,^[^ ^45^, ^46^ ^]^ abundant transgenic lines,^[^ ^47^ ^]^ and rapid development during early embryogenesis.^[^ ^46^ ^]^ More importantly, they share 70% homologous genes with humans,^[^ ^48^ ^]^ which makes them an ideal candidate to explore the cardiac left‐right development under light stimulation. The LRO started to form at the dorsal node of the zebrafish embryo at 8 h post‐fertilization (hpf). As indicated in Figure 1c1, the LRO at 8 somite‐stage (ss) exhibited an ellipse shape with its long axis and short axis ≈75 and 55 µm, respectively (Figure 1c2; Figure S1, Supporting Information). Notably, the LRO undergoes dynamic size changes during embryonic development and reaches maximum dimensions at ≈10 ss, subsequently regressing until it completely disappears during later developmental phases (Figure S2, Supporting Information). Meanwhile, one cilium was clearly observed at the periphery of the LRO, which exhibited an autonomous rotation along the clockwise direction (indicated by the navy arrow in Figure 1c3). Notably, there were tens of cilia in the LRO, whose average length and diameter were 6.5 µm (Figure 1d) and 600 nm (Figure 1e), respectively. In addition to the periphery, the cilia were also observed on the dorsal roof and ventral floor of the LRO (Figure S3, Supporting Information), which exhibits a uniform distribution throughout the LRO. Moreover, their rotation speed *ω* ranged from 2 to 22 rev s^−1^ with an average value of 12 rev s^−1^ (Figure 1f). In addition, the rotation speed varied across different orientations within LRO. For instance, the cilia in the anterior region exhibited a faster rotation than those in the posterior region, while the left‐side cilia rotated faster than those in the right‐side counterparts (Figure S4, Supporting Information). In addition to cilia, the heart of the zebrafish embryo was also characterized. As indicated in Figure 1g, for the zebrafish at 5 days post‐fertilization (dpf), the heart exhibited a clear atrial‐ventricular differentiation with a normal leftward jogging (Figure 1g). Notably, the zebrafish heart experienced a rapid development with significant morphological change during early embryogenesis, i.e., from an S‐shaped curved heart tube to significant atrioventricular differentiation within the period of 2 to 7 dpf (Figure S5, Supporting Information).

### 2.2 Direct Optical Manipulation of Cilia

**Figure 2:**
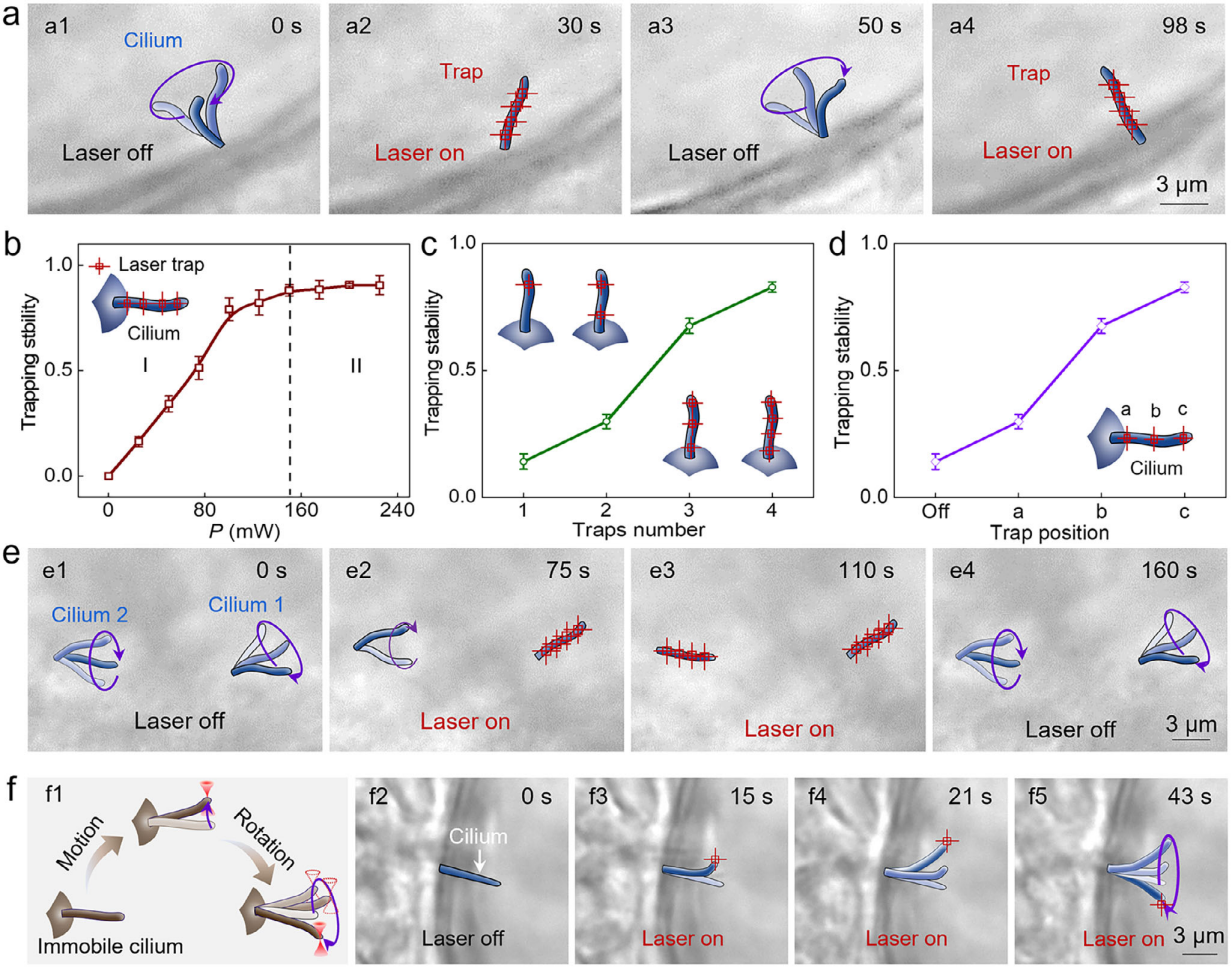
Direct optical manipulation of cilia. a) Optical microscopic image for trapping one motile cilium. b–d) The calculated trapping stability as a function of the laser power (b), optical traps (c), and the exerted position (d). e) Optical microscopic image for simultaneous trapping of two cilia. f) Optical microscopic image for manipulating the immotile cilia.

To validate the manipulation flexibility of optical tweezers on ciliary systems, a stable trapping was conducted first for the motile cilia within LRO. As indicated in **Figure** **2**a1, one cilium was rotating in the clockwise direction with a speed of 2.5 rev/s. After four optical potential wells (OPWs) were exerted on the cilium at *t* = 30 s, it stopped rotating and then remained stationary with its orientation along the predesigned direction (Figure 2a2). By removing OPWs at *t* = 50 s, the rotation was restored immediately (Figure 2a3), and the cilium could be trapped again by introducing laser beams at *t* = 98 s (Figure 2a4; Figure S6, Supporting Information). Moreover, the high spatiotemporal precision of optical manipulation enables the selective trapping of individual cilia, without affecting surrounding cilia (Figure S7 and Movie S1, Supporting Information). Furthermore, the trapping stability *η* was calculated, which was defined as *η* = (*l_free_−l_trap_*)/*l_free_*, where *l_free_* and *l_trap_* were the ciliary rotational amplitude under free oscillation and optical trapping, respectively. As indicated in Figure 2b, the trapping stability was increased with laser power (*P*) due to the enhanced optical force (region I). Once the laser power reached 150 mW, the cilium achieved a stable trapping, after which the trapping stability *η* remained nearly constant (region II). In addition to laser power, the trap stability *η* was also depended on the number and position of optical traps. As indicated in Figure 2c, the trapping stability increased from 14% to 83% as the number of OPWs increased from 1 to 4. Besides, a more stable trapping was achieved by exerting the optical trap at the head position than the middle or tail end, which should contribute to a larger optical force torque (Figure 2d). Crucially, the laser power required for stable trapping depended on cilia rotation speed in LRO (Figure S8, Supporting Information), likely because rapid rotation necessitates stronger optical forces to decelerate cilia for desired immobilization.

**Figure 3:**
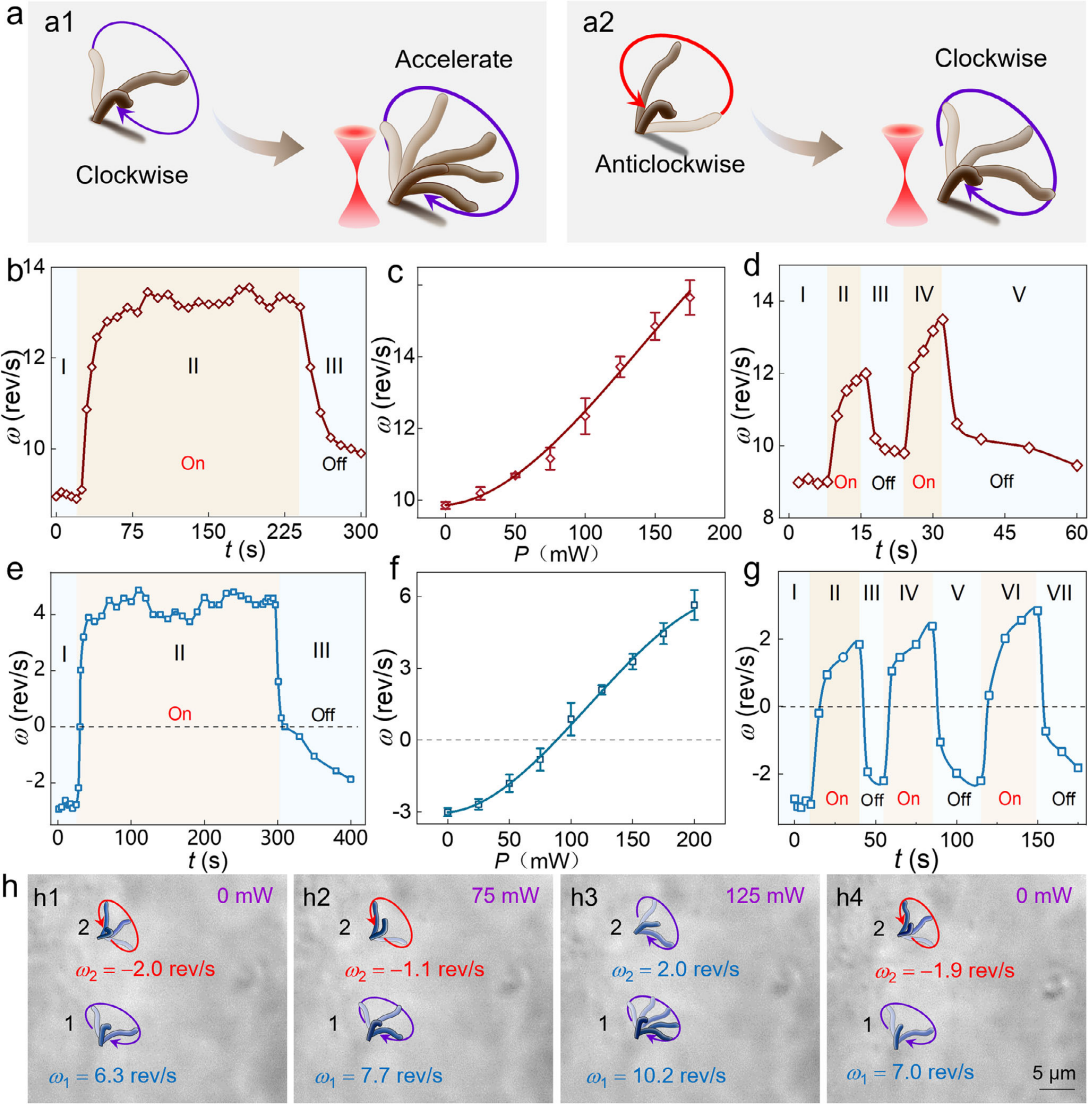
Indirect optical regulation of cilia. a) Schematic diagram of regulating ciliary rotation for the cilia rotating in the clockwise (a1) and anticlockwise direction (a2). b,c) The calculated *ω* as a function of time *t* (b) and laser power *P* (c) for the cilia rotating in the clockwise direction. d) The calculated *ω* during periodic optical modulation for the cilia rotating in the clockwise direction. e,f) The calculated *ω* as a function of time *t* (e) and laser power *P* (f) for the cilia rotating in the anticlockwise direction. g) The calculated *ω* during periodic optical modulation for the cilia rotating in the anticlockwise direction. h) Simultaneous modulation of two cilia which were rotating in opposite direction.

**Figure 4:**
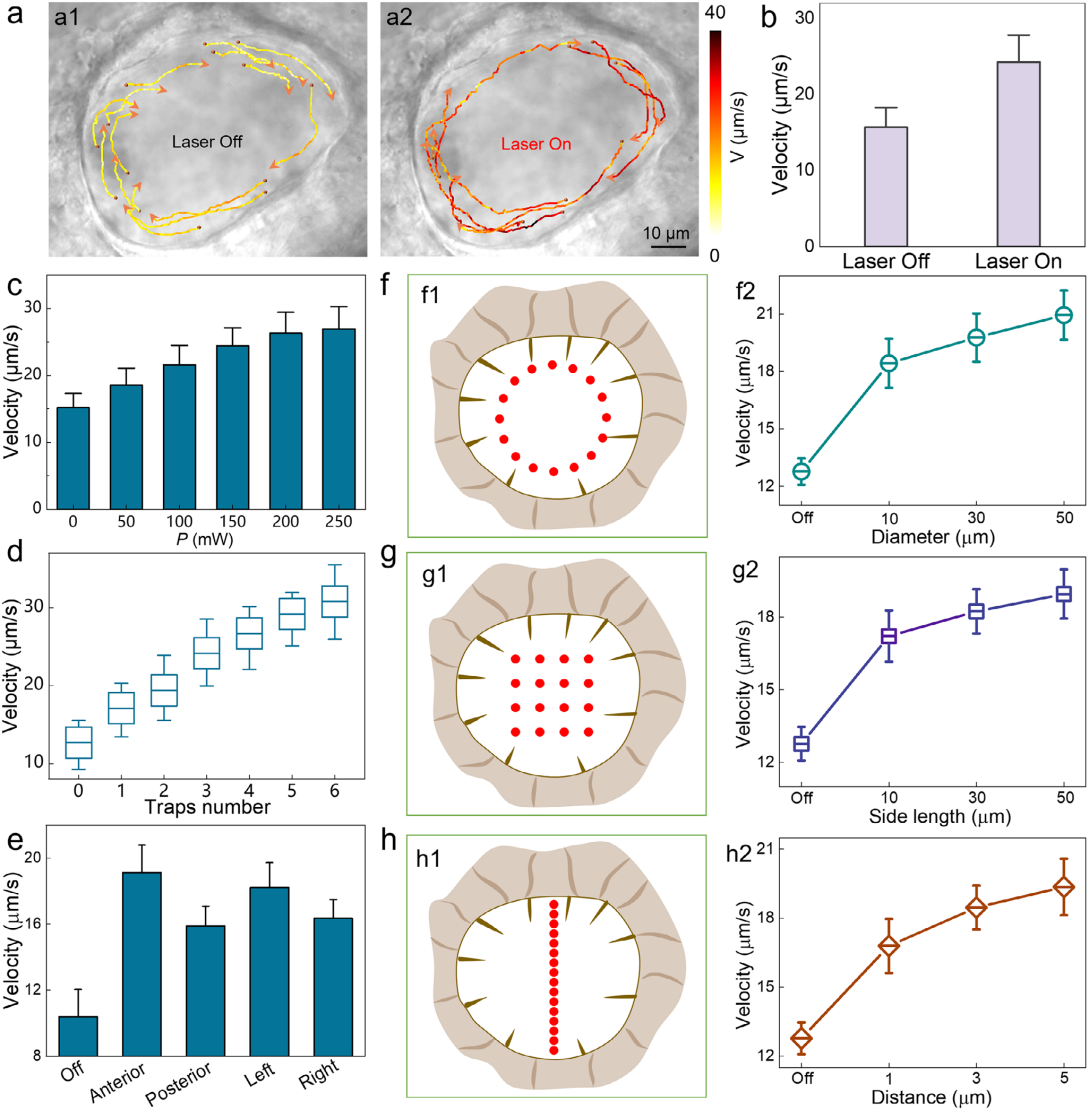
Programmable sculpting of recirculating microflow within LRO. a) The calculated particle motion trajectory to interpret microflow velocity before (a1) and after (a2) laser activation. b) Quantitative analysis of microflow velocity before and after laser activation. c–e) The calculated velocity of recirculating microflow as a function of laser power *P* (c), trap number (d), and irradiation region (e). f–h) Quantitative analysis of microflow modulation under the stimulation of three geometrically configured beam patterns, including circular (f), rectangular (g), and linear (h) arrays.

By introducing more OPWs, multiple cilia could be trapped simultaneously. As indicated in Figure 2e1, two cilia were rotating in the clockwise direction with a speed of 5.0 and 3.5 rev s^−1^, respectively. After introducing four OPWs at *t* = 75 s (*P* = 150 mW), cilium 1 was trapped stably while cilium 2 kept the clockwise rotation (Figure 2e2). Subsequently, four OPWs were exerted on the cilium 2 one by one, under which the rotation amplitude gradually decreased and a stable trapping was eventually reached at *t* = 110 s (Figure 2e3). By removing the laser beam, the two cilia were recovered to the clockwise rotation again (Figure 2e4). The detailed rotational velocities were further characterized to interpret the above experiment process in a quantitative manner (Figure S9, Supporting Information). In addition to the motile cilia, there were some immotile cilia within LRO that could not rotate in the autonomous state. However, they could experience controlled rotational motion upon photonic force application (Figure 2f1). As indicated in Figure 2f2, one cilium was growing in the LRO and remained stationary without the desired rotation. After one OPW was exerted on its end, the cilium was trapped immediately and then exhibited a synchronous motion with OPW (Figure 2f3‐f5). In this way, the desired rotation of the immotile cilium was achieved with a controlled velocity and direction (Movie S2, Supporting Information). Thus, under the action of programmable optical force, both motile and immotile cilia could be manipulated in a flexible manner.

**Figure 5:**
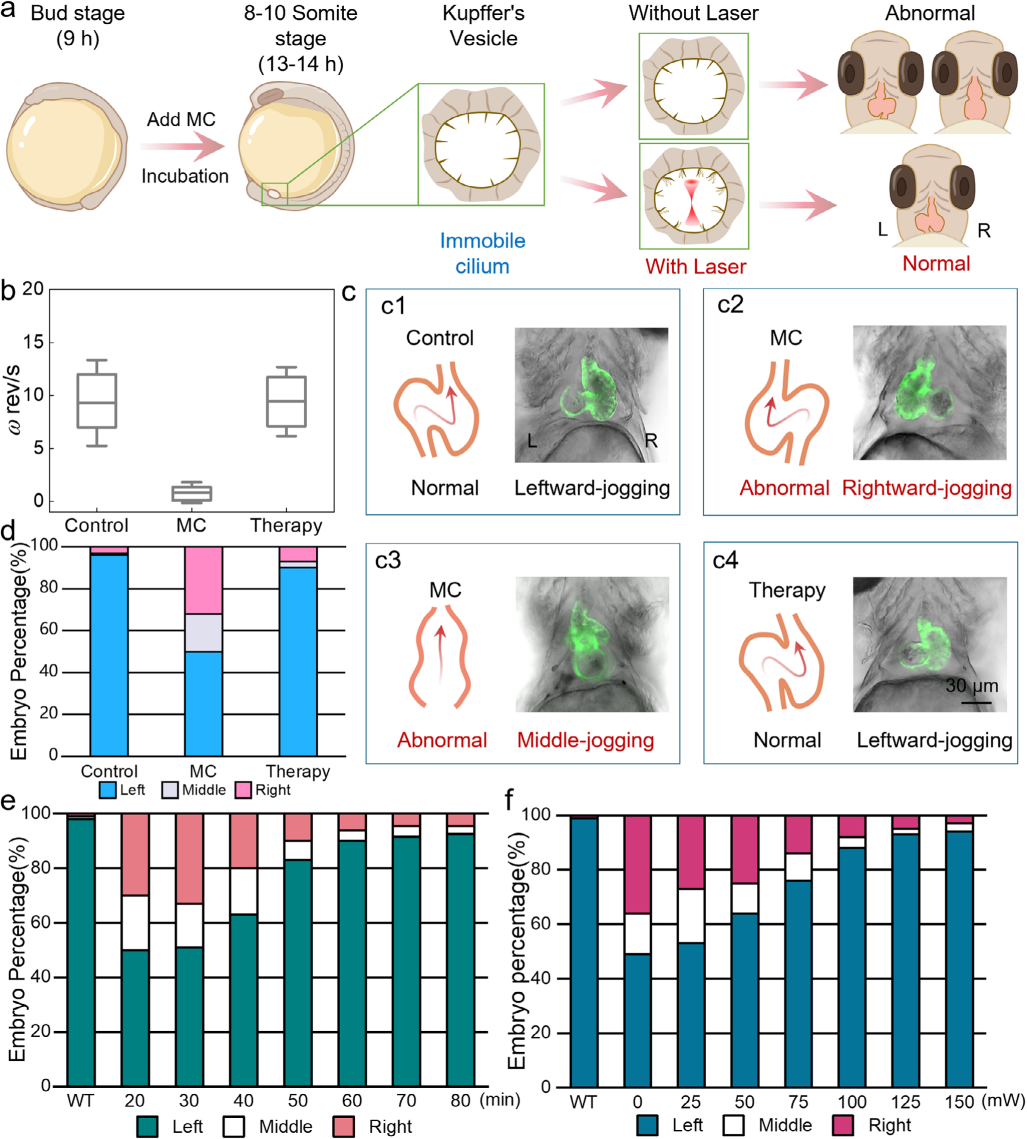
Instructing cardiac left‐right asymmetry development by the Opto‐Bio‐Hydrodynamic platform. a) Experimental design for instructing cardiac development by manipulating cilia within LRO. b) The ciliary rotational speed before and after optical manipulation. c) Schematic diagram and microscopic images of different cardiac jogging directions. d) The cardiac jogging direction before and after laser regulation. e‐f) Quantitative characterization of cardiac development regulatory performance on the modulation duration (e), laser power (f).

### 2.3 Indirect Optical Regulation of Cilia

In addition to direct optical trapping, the cilia could also be regulated in an indirect manner. For this case, the light beam was irradiated onto the hair cell directly, after which the cilia exhibited two different motion behaviors: the cilia rotating in clockwise direction exhibited a significant acceleration (**Figure** **3**a1; Movie S3, Supporting Information), while the cilia rotating in anticlockwise direction showed a gradual deceleration with a reverse acceleration, i.e., transitioning to the clockwise direction (Figure 3a2; Movie S4, Supporting Information). As indicated in Figure 3b, one cilium was first rotating with a velocity of *ω* = 9.0 rev s^−1^ in the clockwise direction (region I). After the laser was turned on at *t* = 25 s, the ciliary motion velocity increased immediately and eventually stabilized at *ω*_max_ = 13.3 rev s^−1^ (region II). This velocity elevation was reversible, as evidenced by the gradually decreased velocity upon the removal of the laser beam (region III). Notably, the achieved *ω*_max_ was increased with the laser power (Figure 3c; Movie S5, Supporting Information), further confirming that the dynamic modulation of ciliary rotation was attributed to the exerted optical stimuli. Furthermore, the ciliary rotation could be modulated in a periodic manner, during which the rotational velocity exhibited an immediate response to the exerted optical stimulation with excellent repeatability (Figure 3d). Notably, the cilia across all monitored regions exhibited a uniform trend of increased clockwise velocity, while the magnitude of velocity enhancement varied with the distance from the OPW (Figure S10, Supporting Information).

Then, the optical regulation of cilia rotating in the anticlockwise direction was investigated. As indicated in Figure 3e, the cilium initially exhibited a rotational velocity of −2.9 rev s^−1^, in which the negative values indicated counterclockwise rotation. Upon laser activation at *t* = 25 s, the rotational velocity progressively transitioned from −2.9 rev s^−1^ (counterclockwise) through zero to +4.6 rev s^−1^ (clockwise), achieving a complete directional reversal. Moreover, the magnitude of velocity modulation increased with the laser power, with rotational direction switching, i.e., from counterclockwise to clockwise, triggered at *P* = 90 mW (Figure 3f). Remarkably, this bidirectional switching behavior demonstrated high reproducibility across multiple cycles (Figure 3g). Besides, the ciliary speed exhibited an inverse correlation with irradiation distance (Figure S11, Supporting Information) and a linear increase with the number of OPWs (Figure S12, Supporting Information), which was ascribed to the increased spatial attenuation and enhanced stimulation by the cumulative photon absorption, respectively.

Building upon these findings, a simultaneous optical regulation was conducted for two cilia that were rotating along the opposed directions. As indicated in Figure 3h1, two cilia, i.e., cilium 1 and 2, exhibited an initial rotational velocity of +6.3 (clockwise) and −2.0 rev s^−1^ (counterclockwise), respectively. Upon laser activation at *P* = 75 mW, their velocities were regulated to +7.7 and −1.1 rev s^−1^, respectively (Figure 3h2). Subsequently, the laser power was increased to 125 mW, under which both cilia achieved synchronized clockwise rotation with the velocity of +10.2 and +2.0 rev s^−1^, respectively (Figure 3h3). This bidirectional control was also reversible, as evidenced by the gradual restoration of their original rotational states after removing the laser beam (Figure 3h4). Therefore, the indirect optical stimulation can act as a robust technology to enable precise bidirectional control over both rotational velocity and direction in ciliary systems.

### 2.4 Programmable Sculpting of Recirculating Microflow within LRO

The indirect ciliary manipulation operates through photothermal activation of calcium channels in hair cells, which will trigger intercellular signaling cascades via connexin‐26 gap junctions.^[^ ^49^, ^50^ ^]^ Consequently, all motile cilia are synchronously regulated, and this coordinated ciliary beating will sculpt the recirculating microflow within LRO in a programmable manner. As shown in **Figure** **4**a, a characteristic recirculating flow was observed in LRO, as evidenced by the synchronous clockwise rotation of endogenous particles with an average velocity of *V* = 16 µm s^−1^. Upon laser irradiation on the hair cell, the accelerated ciliary beating frequency facilitated viscous stress generation, expediting the flow velocity to 24.5 µm s^−1^ (Figure 4a2,b). As shown in Figure 4c, the average velocity of recirculating flow within LRO exhibited a positive correlation with laser power (15 ± 2.5 µm s^−1^ at 0 mW versus 27 ± 4 µm s^−1^ at 250 mW). This trend remained consistent when increasing OPW configuration, where six optical traps enhanced microflow velocity from 12.7 ± 3 µm s^−1^ to 30.8 ± 6 µm s^−1^ (Figure 4d). These parametric dependencies establish a quantitative framework to sculpt the microflow architecture through coordinated control of optical power density and optical trap number.

Besides, spatiotemporal analysis uncovered region‐specific optical modulation efficacy across the LRO. As indicated in Figure 4e, anterior photo‐stimulation induced significantly greater flow acceleration compared to posterior regions, while left lateral stimulation outperformed right counterparts. This spatial heterogeneity highlights the necessity to incorporate spatial mapping into the stimulation algorithm to optimize intervention precision, particularly for developmental disorders involving left‐right axis specification.

To elucidate the coupling principle between beam mapping and hydrodynamic modulation, the regulatory efficacy was systematically investigated for various beam patterns on the recirculating flow. By programming same sixteen optical traps, three distinct topological patterns were engineered: concentric circular arrays (diameter: 10–50 µm, Figure 4f), rectangular grids (side length: 10–50 µm, Figure 4g), and linear arrays with tunable inter‐trap spacing (distance: 1–5 µm, Figure 4h). Notably, systematic geometric scaling induced enhanced modulation of recirculation microflow, which reconfirmed the flexible sculpture on the recirculating microflow by designing the beam pattern in a real‐time programmable manner. This Opto‐Bio‐Hydrodynamic coupling demonstrates precise spatiotemporal control over developmental fluid mechanics through optical regulation of ciliary rotation. The sculpted microflow can further promote calcium activity and modulate asymmetric gene expression, thus enabling the instruction of left‐right asymmetric development of embryos.

### 2.5 Instructing Cardiac Left‐Right Asymmetry Development by Opto‐Bio‐Hydrodynamic Platform

To investigate the instruction of cardiac left‐right asymmetry development by cilia modulation, zebrafish embryos at 9 hpf were co‐incubated with methylcellulose (MC) to establish a ciliary rotation deceleration model (**Figure** **5**a). As a thickening material, MC could increase the fluid viscosity within the LRO while inducing a transient Ca^2^⁺ decrease at the ciliary base, thus leading to elevated fluid resistance and weakened intrinsic driving forces. These synchronized effects ultimately resulted in a significant reduction in ciliary motion velocity and frequency. After 4 h of co‐incubation with MC, the ciliary length and diameter remained consistent with those in the untreated control group (Figure S13, Supporting Information). However, their rotational speed significantly decreased from 10 to 0.7 rev s^−1^ (Figure 5b). Concurrently, the flow velocity of recirculating microfluid within the LRO was also reduced (Figure S14, Supporting Information). Consequently, the embryos exhibited abnormal cardiac orientation during subsequent development (Movie S6, Supporting Information), transitioning from the normal leftward‐jogging configuration (Figure 5c1) to pathological rightward‐jogging (Figure 5c2) and middle‐jogging (Figure 5c3) phenotypes. As shown in Figure 5d, compared to 4% in the control group, MC‐treated embryos exhibited a 50% incidence of cardiac developmental abnormalities by 5 dpf, among which the middle‐jogging and rightward‐jogging phenotypes accounted for 20% and 30%, respectively. These results confirm that aberrant ciliary motility critically disrupts normal cardiac development, ultimately inducing cascading organogenesis defects.

However, optical manipulation of rotationally impaired cilia can gradually restore normal cardiac development. As indicated in Figure 5b, application of optical intervention successfully restored ciliary rotational speed to physiological levels. Meanwhile, almost full recovery of recirculatory flow patterns within the LRO was observed (Figure S14, Supporting Information). Benefited from this, zebrafish cardiac development was effectively rescued (Figure 5c4), i.e., reinstating the left cardiac jogging direction. As indicated in Figure 5d, the proportion of normally developed hearts significantly increased from 50% to 92% after a continuous optical manipulation over 1 h within the LRO, thereby validating the remarkable potential of optical modulation in rectifying cardiac laterality defects.

Furthermore, the relationship was investigated in a quantitative manner between cardiac laterality regulation and optical manipulation parameters, including laser power and manipulation duration (*t*). As shown in Figure 5e, when the intervention duration was *t* ≤ 30 min, optical modulation failed to correct cardiac developmental abnormalities, with cardiac jogging remaining to exhibit left‐right randomization. However, as intervention duration increased to 40 min, optical manipulation began to systematically influence cardiac laterality patterning and rescued developmentally abnormal hearts. Specifically, the proportion of normally developed hearts, i.e., leftward jogging, progressively increased to 63%, 83%, and 90% for the manipulation durations of 40, 50, and 60 mins, respectively. The manipulation duration beyond 60 min might yield a diminishing return, for which the normal cardiac development rates were 92% and 93% for the cases of 70 and 80 min, respectively. Since the photodamage threshold was associated with prolonged irradiation, 60 min was selected as the optimal optical manipulation duration to balance therapeutic efficacy with biosafety constraints.

In addition to manipulation duration, the regulatory efficacy on cardiac laterality defects was also quantified for laser power. As indicated in Figure 5f, the proportion of cardiac developmental abnormalities remained at 53% for the case of *P* = 25 mW, indicating insufficient therapeutic intervention. As the power increased to 50 mW, the corrective performance improved significantly, i.e., achieving 64% normal cardiac development. Further enhancement was observed at 75, 100, and 125 mW, with normal cardiac development rates progressively rising to 76%, 88%, and 91%, respectively. The regulation performance gradually plateaued once the exerted laser power was larger than 125 mW. The above results demonstrate that cardiac developmental abnormalities can be substantially regulated under defined optical manipulation, i.e., 60‐min regulation duration with the power of 125 mW. Therefore, optical modulation of LRO ciliary dynamics effectively counteracts cilia‐driven cardiac malformations. This approach establishes a novel conceptual framework for elucidating pathogenesis mechanisms in congenital heart disease through ciliary functional analysis, which might advance early‐stage preventive strategies and develop innovative therapeutic modalities targeting ciliopathy‐associated disorders.

## 3 Discussion

In this work, an Opto‐Bio‐Hydrodynamic platform was constructed to regulate the ciliary motility patterns through programmable optical force with the aim of instructing the cardiac left‐right asymmetric development. The results demonstrated that the optical intervention could real‐time modulate the ciliary beating pattern, under which the inner recirculating microflow could be sculptured in a programmable manner and then exploited to rescue the cardiac positioning anomalies from 50% to 93% in pharmacological models. Notably, optical trapping of single cilia cannot override the collective flow dynamics essential for symmetry breaking when all cilia remain functional (Figure S15, Supporting Information). Strikingly, however, in zebrafish that lack motile cilia, targeted optical reactivation of single cilia was found to be sufficient to rescue and reverse cardiac situs in zebrafish embryo.^[^ ^17^ ^]^ Compared to other ciliary manipulation techniques, the reported strategy exhibited an improved biocompatibility by avoiding the elaborate injection of exogenous magnetic particles, preventing the acoustic wave interference, and circumventing potential physical damage caused by the fiber probe.^[^ ^30^, ^31^, ^32^ ^]^ Moreover, the reported strategy can also be exploited to manipulate the cilia within the inner ear, with their deflection angle and motion velocity regulated in a spatiotemporally controlled manner (Figure S16, Supporting Information). These results confirm the proposed optical manipulation strategy enables precise targeting of cilia across distinct zebrafish microenvironments, thereby establishing a versatile platform for investigating the physiological functions and mechanotransduction mechanisms of diverse ciliary subtypes. Furthermore, programmable scanning of beam patterns enables simultaneous manipulation of multiple cilia and sculpturing the recirculating microflow within LRO, thereby achieving a precise intervention of cardiac left‐right asymmetry development in a flexible and controlled manner.

Crucially, the safety concerns should be addressed due to the unavoidable water absorption and potential heating. Underemployed optical power levels for LRO ciliary manipulation, comparative analysis revealed equivalent survival rates, heart rate, and spatial patterning of cardiac development (Figure S17, Supporting Information), thereby reconfirming the high biocompatibility of the proposed Opto‐Bio‐Hydrodynamic platform. That might contribute to that all optical interventions were performed at a wavelength of 1064 nm, leveraging its relatively weak scattering and absorption in the tissue of zebrafish embryos as well as low photothermal risks. The maximum laser power was 250 mW, under which a maximum temperature rise of 1.6 °C could be generated in vitro.^[^ ^50^, ^51^ ^]^ However, the agarose embedding matrix and embryonic perivitelline fluid could attenuate the actual light power delivered to the LRO, thus reducing thermal exposure. This result validates the operational safety of the proposed optical methodology for further in vivo applications. In addition, quantitative thresholds remain to be explored for the ciliary rotational velocity required for rescuing visceral symmetry abnormality. Future studies should establish these biomechanical benchmarks to enable AI‐driven monitoring platforms capable of screening embryos with aberrant ciliary dynamics, thereby enabling real‐time modulation of cardiogenesis abnormality. Furthermore, the spatiotemporal coupling between ciliary motion and Nodal signaling gradients has not been dynamically mapped, which can be conducted by introducing fluorescent reporter genes to achieve synchronous imaging of gene expression and mechanical signals, thereby gaining a more comprehensive vision to understand this complex process.

## 4 Conclusion

In conclusion, this study establishes an Opto‐Bio‐Hydrodynamic platform for spatiotemporal instruction of cardiac left‐right asymmetry by reorganizing ciliary dynamics with programmable near‐infrared light. Experimental results demonstrated that direct optical trapping of cilia could stably suppress their beating, while indirect optical regulation enabled systemic modulation across all cilia within LRO, thereby sculpturing the recirculating microflow in a controlled manner. Benefited from the enhanced recirculating microflow, the drug‐induced cardiac development abnormality could be rescued in an efficient manner during the critical embryonic developmental window. These results mechanistically bridge ciliary biomechanics, flow‐mediated signaling gradients, and asymmetric organogenesis, offering quantitative thresholds for therapeutic intervention. By enabling real‐time correction of ciliopathy‐associated developmental errors, this work might provide new insights into the mechanistic understanding of congenital heart defects and other developmental disorders. Furthermore, it establishes opto‐biomechanical modulation as a paradigm‐shifting strategy for improving congenital heart defects, bearing direct implications for early embryonic intervention strategies and mechanobiological therapeutics targeting visceral asymmetry disorders.

## 5 Experimental Section

### Experiment Setup

The experiment setup was constructed around a scanning optical tweezer system (SOTs). The laser beam at a wavelength of 1064 nm was first interacted with an acoustic‐optic deflector to enable a programmable modulation, after which the laser beam was expanded by the beam expander, reflected upward through the dichroic mirror, and then refocused onto the LRO through 60× water immersion inverted objective (CFI Apo, NA = 1.0). Moreover, a halogen light source was used to illuminate the sample from the top while one LED source was integrated to enable a desired fluorescence excitation. The filtrated bands were 465–495 nm, 540–580 nm, and 590–650 nm for acquiring blue, green, and red fluorescence, respectively. The experiment process was monitored through a charge‐coupled device camera and displayed on the computer screen for real‐time monitoring, image capture, and video recording.

### Animal Husbandry

The experimental animals utilized in this study included the AB strain and the cardiac‐specific transgenic cmlc2: EGFP lines. Embryos were obtained from natural spawning and then cultured according to standard protocols.^[^ ^52^ ^]^ Meanwhile, the development stages were determined based on the criteria established by Kimmel et al.^[^ ^53^ ^]^ All procedures involving zebrafish were reviewed and approved by the Experimental Animal Ethics Committee of Jinan University, ensuring adherence to institutional guidelines.

### Embryo Immobilization

Low‐melting‐point agarose (1% w/v) was carefully melted in a water bath and subsequently allowed to cool down to room temperature. Embryos at designated developmental stages were skillfully placed on glass slides and gently embedded within the prepared agarose matrix. Employing fine forceps, the embryos were meticulously positioned to ensure precise alignment of the Kupffer's vesicle with the microscope objective, facilitating optimal visualization. After completing the ciliary manipulation, the embryos were extracted from agarose and transferred to embryo medium for further cultivation at a controlled temperature of 28.5 °C. This process was maintained until 5 dpf, when the cardiac looping was thoroughly assessed for any developmental anomalies or deviations from normalcy.

### Quantification of Ciliary Rotational Speed in KV

The ciliary rotation was first recorded through a high‐speed camera mounted on an inverted microscope. After that, the rotation velocity was quantified by measuring the duration time to complete 10 rotation cycles. To ensure statistical reliability, speed sampling was repeated at 5‐second intervals, with 5 measurements per cilium averaged to determine mean rotation speed.

### Particle Image Velocimetry

Native particles within the KV vesicle were tracked at a rate of 50 frames per second. By using the Manual Tracking plugin in ImageJ, the point‐to‐point tracks can be realized to achieve the detailed motion trajectory for the particles in a quantitative manner. Based on this, instantaneous velocities were computed through particle trajectory analysis, and meanwhile, velocity vectors were accurately mapped to their corresponding spatial coordinates utilizing custom‐written MATLAB scripts.

### Ciliary Dysfunction Model

In order to establish the abnormal model of ciliary rotation, embryos at 9 hpf were incubated with 1% (w/v) solution of methylcellulose for 4 h. The treated embryos were developed to the 8‐somite stage and then delicately subjected to optical manipulation using bright‐field microscopy. After laser intervention, embryos were returned to methylcellulose‐containing medium for continued culture.

## Conflict of Interest

The authors declare no conflict of interest.

## Supporting information



Supporting Information

Supplemental Movie 1

Supplemental Movie 2

Supplemental Movie 3

Supplemental Movie 4

Supplemental Movie 5

Supplemental Movie 6

## Data Availability

The data that support the findings of this study are available from the corresponding author upon reasonable request.
