# Supplementary material for: An Opto‐Bio‐Hydrodynamic Platform for Instructing Cardiac Left‐Right Asymmetry Development
Source: Adv Sci (Weinh). 2025 Aug 18;12(42):e12368. doi: 10.1002/advs.202512368 (PMC12622494; doi:10.1002/advs.202512368)
Supplement: Supplementary file 1 — Supporting Information [file ADVS-12-e12368-s003.docx]

Supporting Information

An Opto-Bio-Hydrodynamic platform for instructing cardiac left-right asymmetry development

Haifeng Qin^1^, Xiaoshuai Liu^2^*, Yufeng Lin^1^, Guangyi Yang^1^, Haojiang Ren^1^, Mingyuan Cao^1^, Zhenheng Jiao^1^, Baojun Li^1*^, Xianchuang Zheng^1*^

^1^Guangdong Provincial Key Laboratory of Nanophotonic Manipulation, Institute of Nanophotonics, College of Physics & Optoelectronic Engineering, Jinan University, Guangzhou 511443, China.

^2^School of Physics and Materials Science, Guangzhou University, Guangzhou 510006, China.

**E-mail**: lxshuai@gzhu.edu.cn (X. L.), baojunli@jnu.edu.cn (B. L.) or xczheng@jnu.edu.cn (X. Z.)

**Table of Contents**

**Figure S1**. Dorsal view and lateral view of the zebrafish embryo LRO.

**Figure S2.** Optical micrographs of the LRO across different development stages in vivo.

**Figure S3.** Optical micrographs of cilia located at the dorsal roof and ventral floor of LRO.

**Figure S4**. Rotation speeds of cilia at various orientations within the LRO.

**Figure S5**. Optical images of zebrafish heart development at different stages.

**Figure S6**. The calculated rotational speed *ω* as a function of *t* during optical trapping of one motile cilium.

**Figure S7**. Optical trapping of the target cilium 1 by sequential application of optical traps.

**Figure S8.** Quantitative characterization of optical trapping power on cilia rotation speed.

**Figure S9**. Quantitative analysis of rotational speed *ω* when two cilia were trapped.

**Figure S10.** Spatial mapping of ciliary rotation variations during indirect optical regulation.

**Figure S11**. The calculated *ω* as a function of the spatial distribution of OPWs.

**Figure S12**. The calculated *ω* as a function of the number of OPWs.

**Figure S13.** Quantitative analysis of ciliary length, diameter and rotational speed in embryo LRO after incubation with methylcellulose.

**Figure S14.** The flow velocity of recirculating microfluid within the LRO.

**Figure S15.** Quantitative characterization of optically manipulated single cilium on cardiac looping directionality.

**Figure S16**. Optical manipulation of the cilia within the inner ear.

**Figure S17**. Biosafety characterization of the Opto-Bio-Hydrodynamic platform.

1. **Dorsal view and lateral view of the zebrafish embryo LRO.**


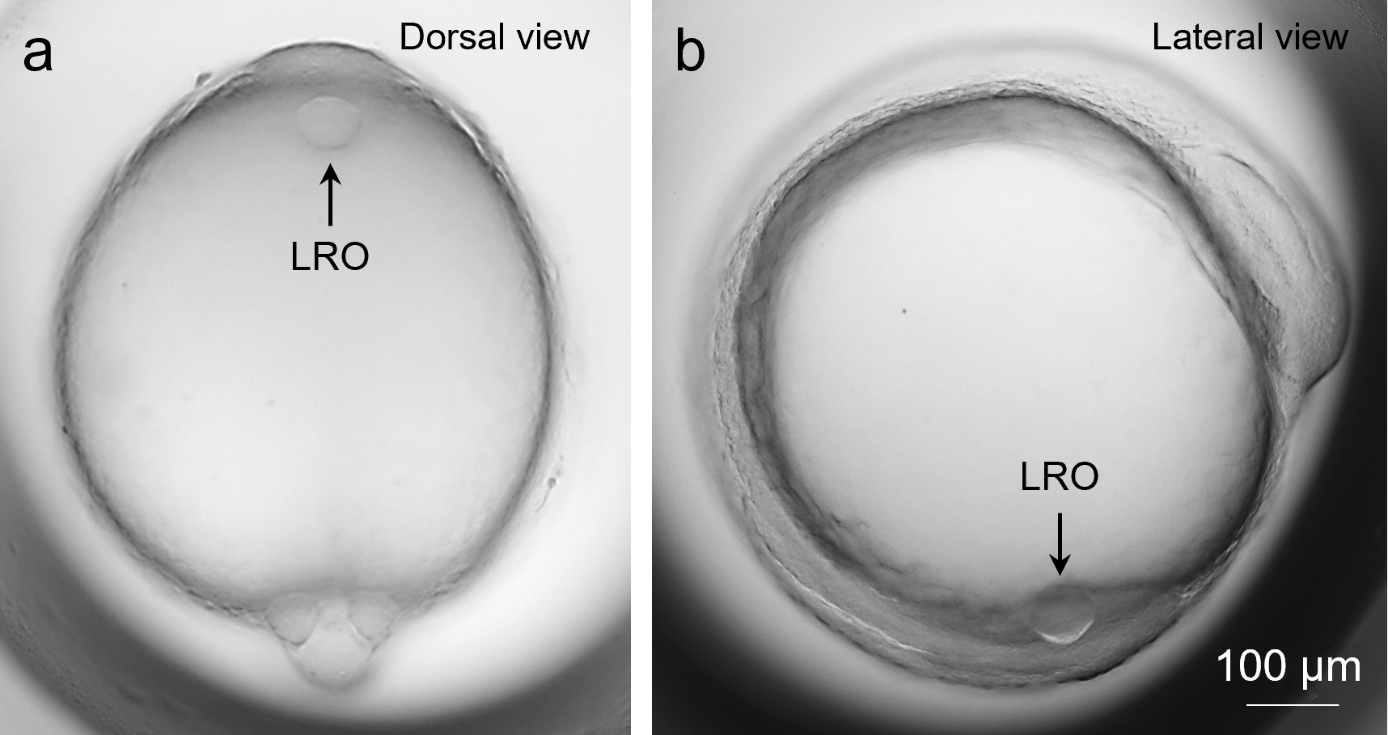


**Figure S1**. Dorsal view (a) and lateral view (b) of the zebrafish embryo LRO. The LRO in the dorsal view exhibits a bright field of view, while the LRO in the lateral view shows a dark shadow which might disturb the real-time characterization of cilia. Therefore, the LRO was observed from the dorsal view rather than the lateral view.

1. **Optical micrographs of the LRO across different development stages in vivo.**


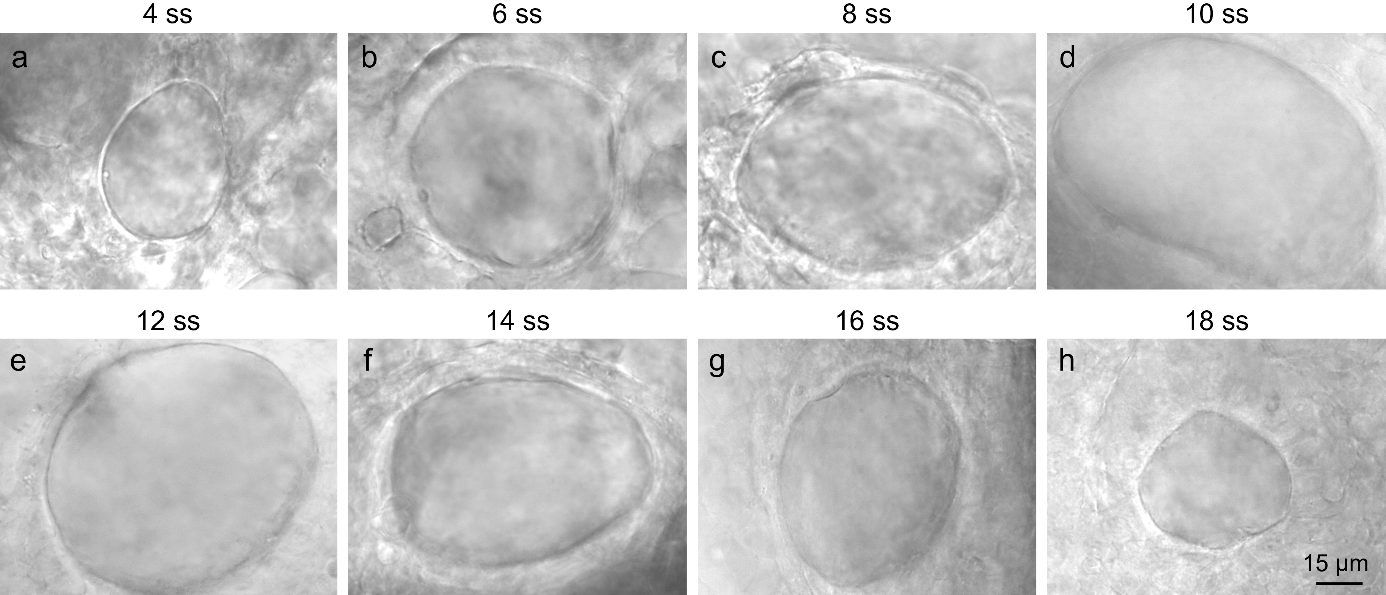


**Figure S2**. Optical micrographs of the LRO across different development stages in vivo.

1. **Optical micrographs of cilia located at the dorsal roof and ventral floor of LRO.**


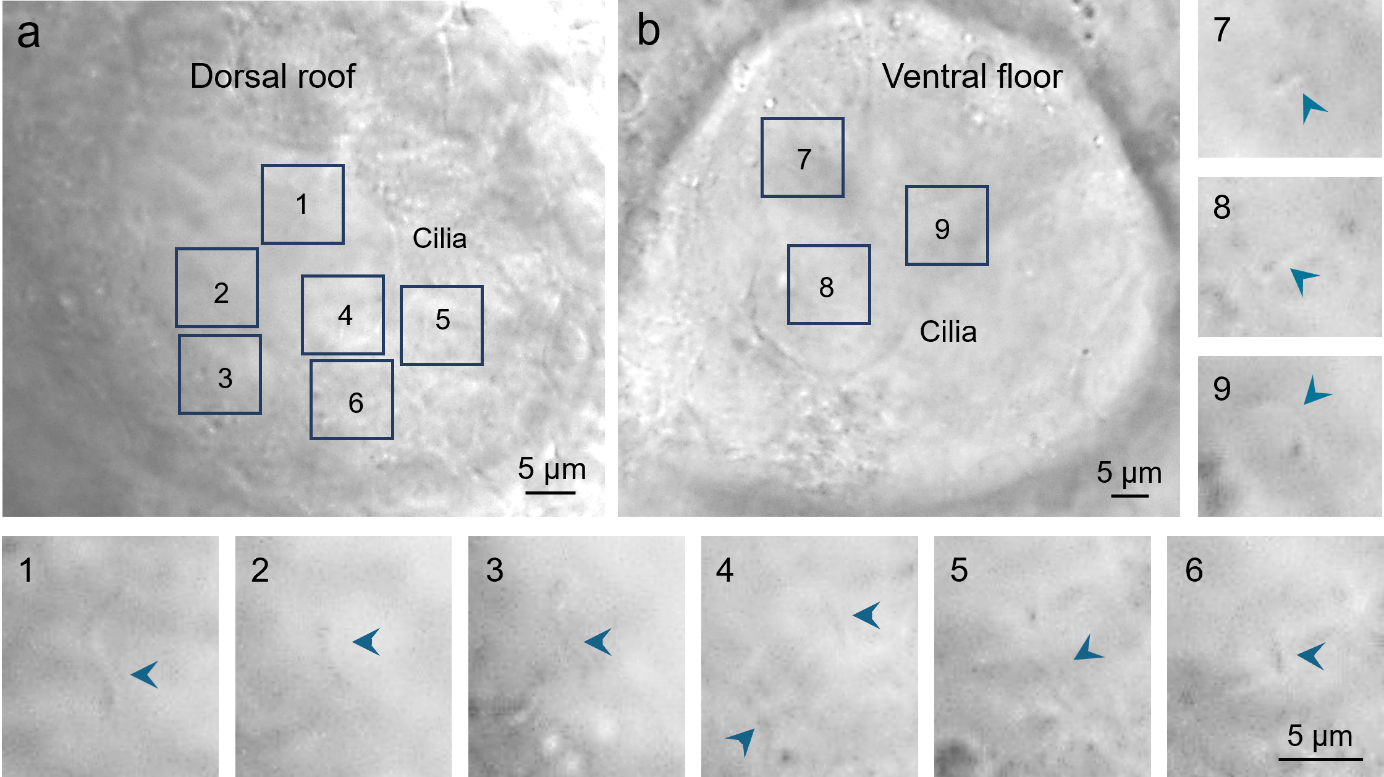


**Figure S3**. Optical micrographs of cilia located at the dorsal roof (**a**) and ventral floor (**b**) of LRO.

1. **Rotation speeds of cilia at various orientations within the LRO.**


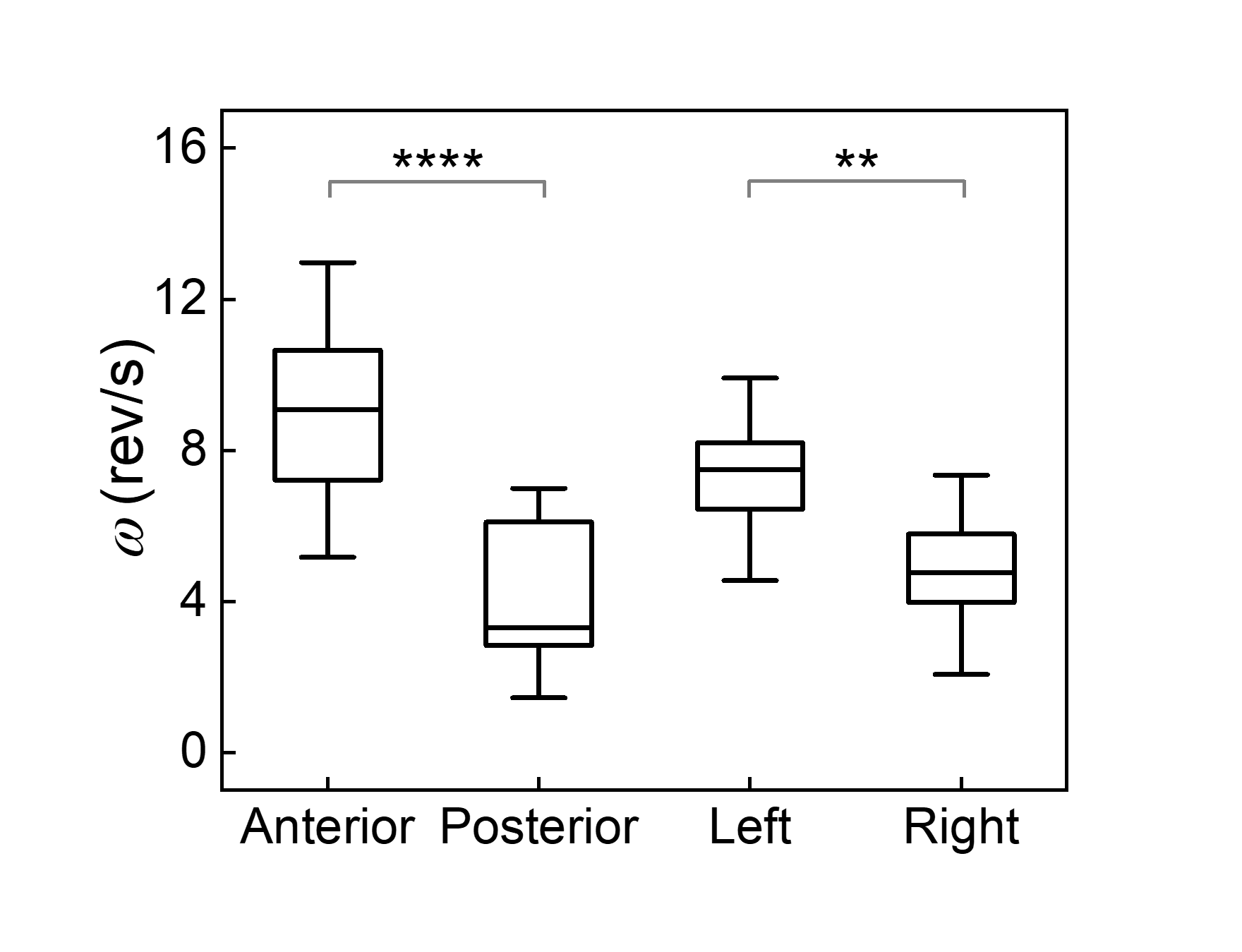


**Figure S4**. Rotation speeds of cilia at various orientations within the LRO.

1. **Optical images of zebrafish heart development at different stages.**


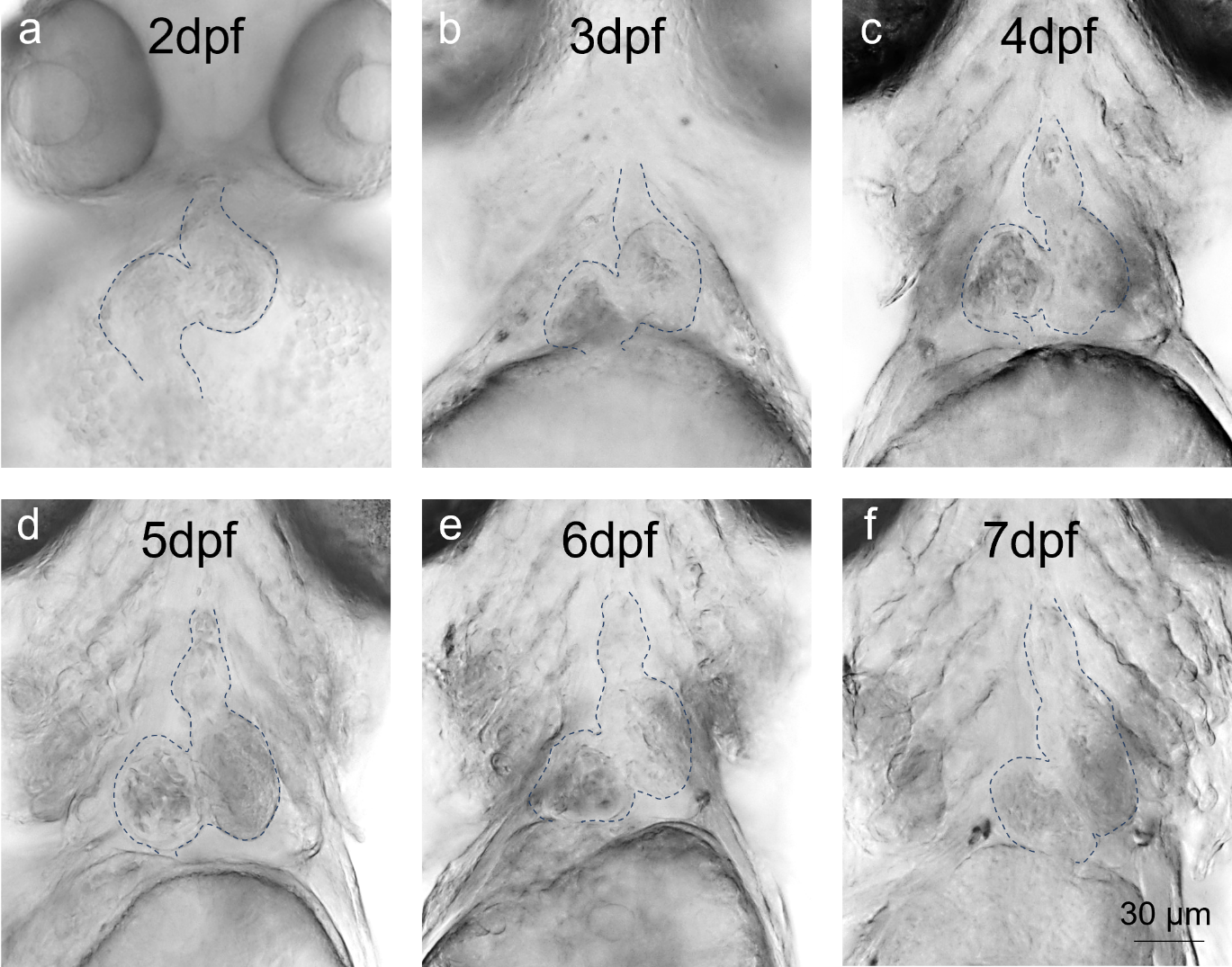


**Figure S5**. Optical images of zebrafish heart development at different stages.

1. **The calculated rotational speed *ω* as a function of *t* during optical trapping of individual motile cilium.**


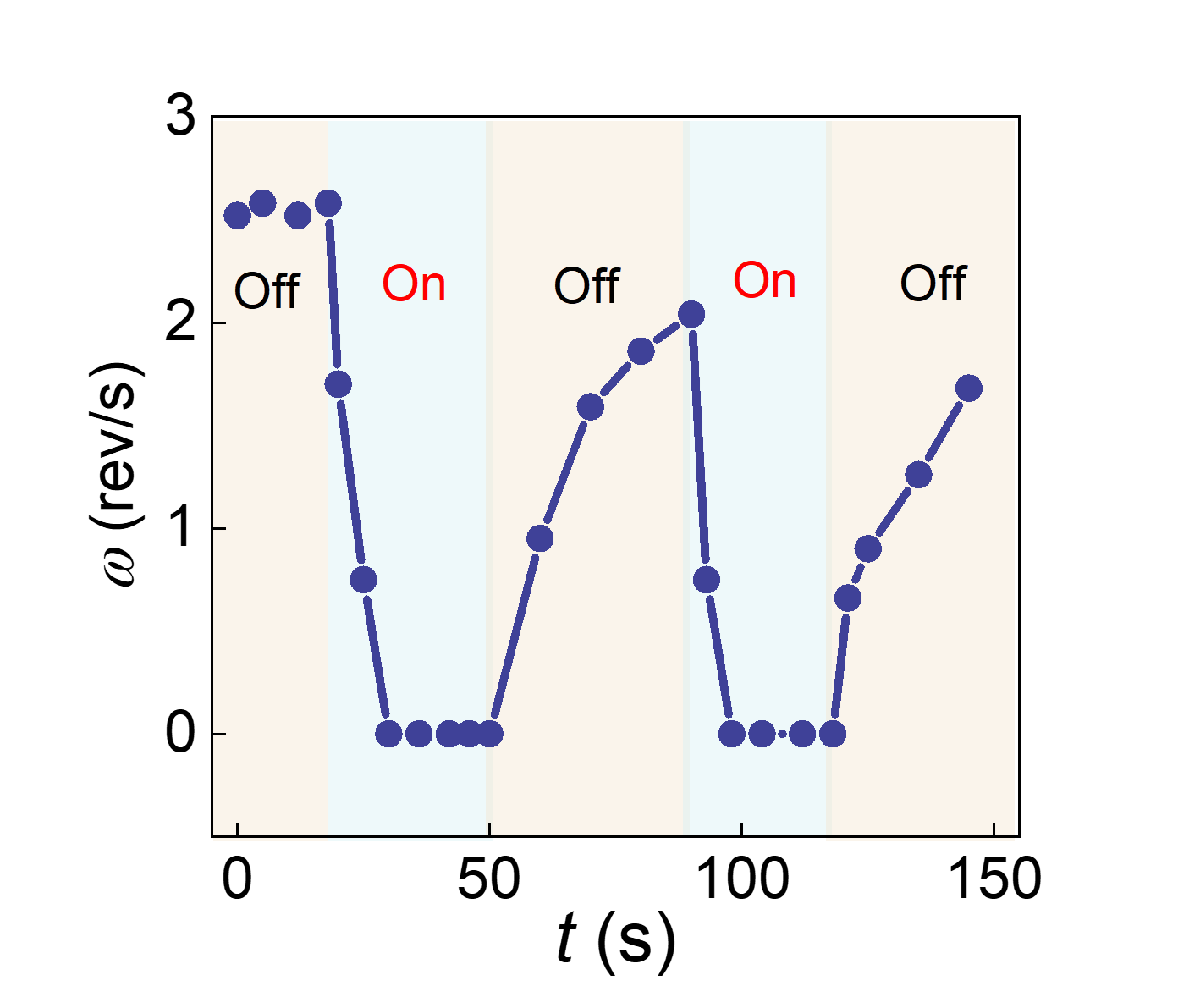


**Figure S6**. The calculated rotational speed *ω* as a function of *t* during optical trapping of individual motile cilium. Notably, *ω* immediately decreased from 2.58 to 0 rev/s after the laser was turned on at *t* = 30 s. However, it exhibited a gradual increase upon removing the laser beam, and was then restored to 2.04 rev/s at *t* = 90 s.

1. **Optical trapping of the target cilium 1 by sequential application of optical traps.**

**
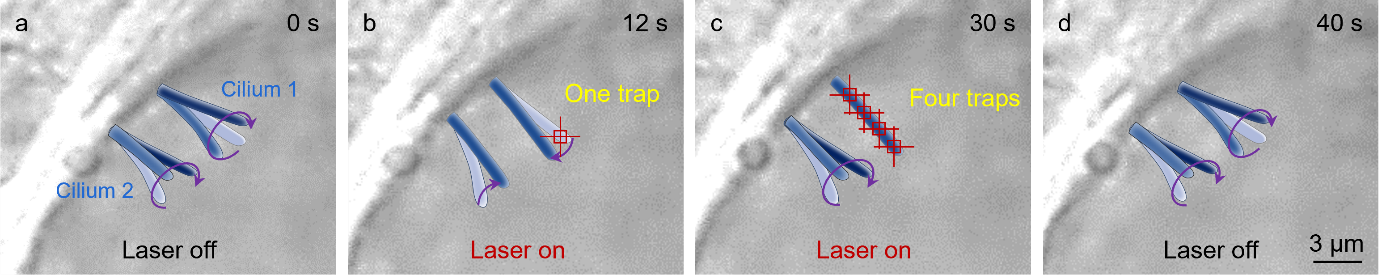
**

**Figure S7**. Optical trapping of the target cilium 1 by sequential application of optical traps. When the OPW was exerted one by one, the cilium 1 exhibited a gradual deceleration until it was trapped stably. In contrast, the nearby cilium 2 remained its dynamic rotation, thus enabling the targeted trapping of individual cilium and confirming the stable trapping was induced by the optical force rather than spontaneous behavior.

1. **Quantitative characterization of optical trapping power on cilia rotation speed.**


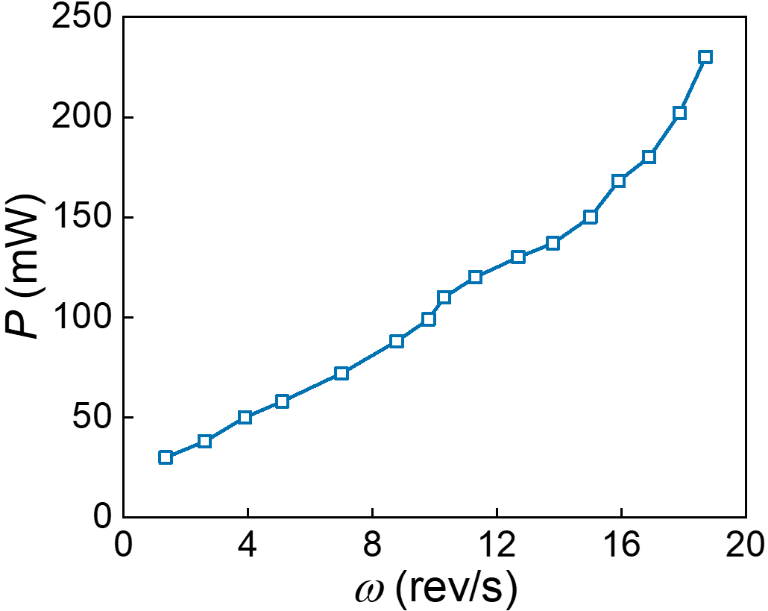


**Figure S8**. The required laser power for stable ciliary trapping as a function of rotational velocity.

1. **Quantitative analysis of rotational speed *ω* when two cilia were trapped.**

**
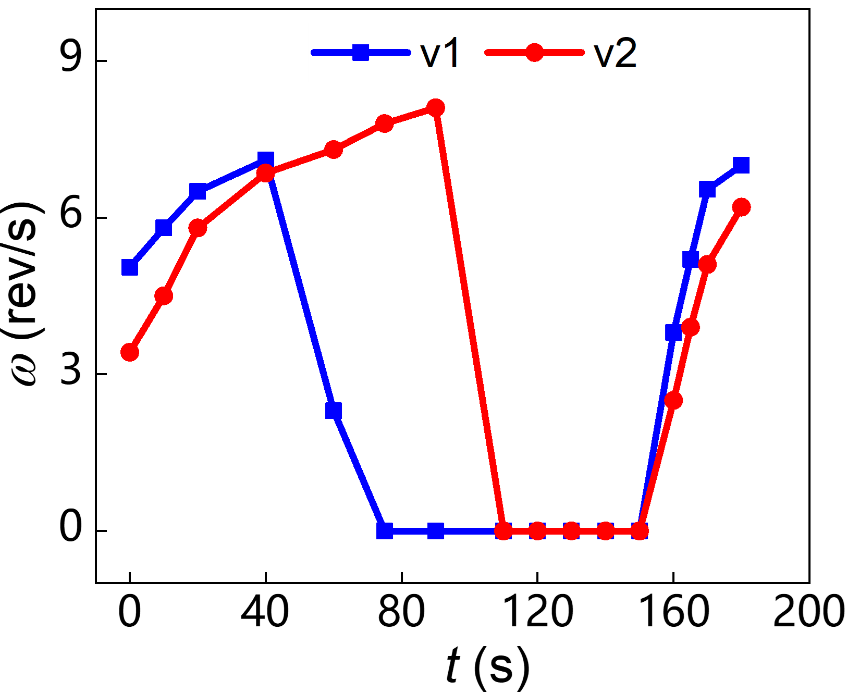
**

**Figure S9**. Quantitative analysis of rotational speed *ω* when two cilia were trapped.

1. **Spatial mapping of ciliary rotation variations during indirect optical regulation.**


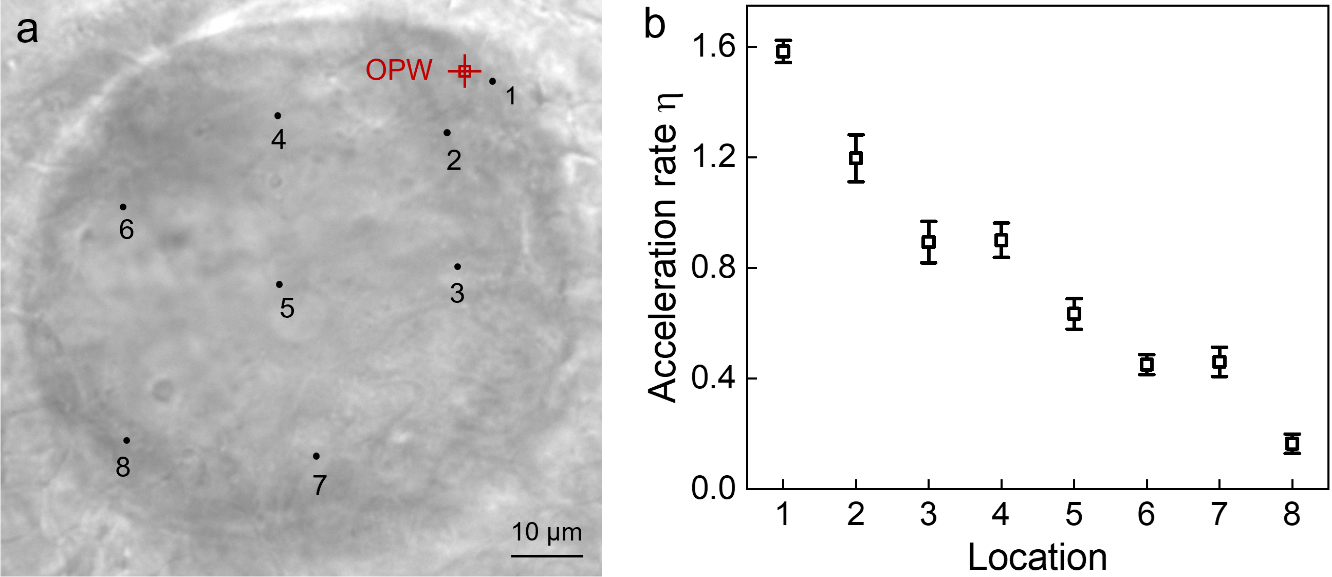


**Figure S10**. Spatial mapping of ciliary rotational speed changes during indirect optical regulation. During the indirect optical regulation, the cilia across all monitored regions exhibited a uniform trend of increased clockwise velocity. However, the magnitude of velocity enhancement varied with the distance from the OPW. To quantify this spatial heterogeneity, the acceleration rate *η* was calculated for the cilia originating from distinct regions, which was defined as *η* = (*ω*_2_ −*ω*_1_) /*ω*_1_, where *ω*_1_ and *ω*_2_ respectively represent pre-stimulation and post-stimulation rotational speeds. Notably, *η* decreased progressively with increased distance from the OPW, with a maximum and minimum magnitude of 1.6 and 0.16 at position 1 and position 8, respectively. The observed results might be attributed to position 1’s proximity to the OPW, where minimized spatial attenuation results in maximal localized photon energy density, thus generating an enhanced optothermal activation of calcium channels in hair cells.

1. **The calculated *ω* as a function of the spatial distribution of OPWs.**

**
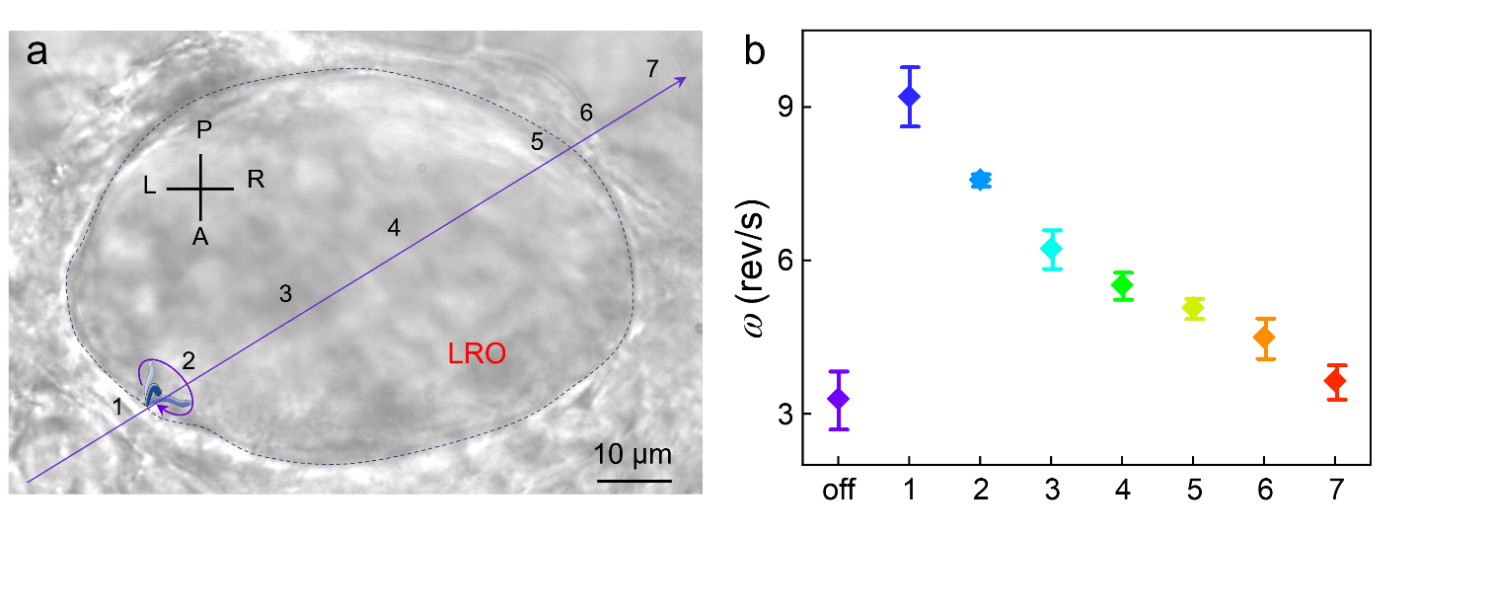
**

**Figure S11.** The calculated *ω* as a function of the spatial distribution of OPWs. The laser beam was sequentially exerted on seven positions and the corresponding ciliary rotation velocity was calculated. The results indicated that the ciliary speed exhibited an inverse correlation with irradiation distance and achieved a maximum value of *ω* = 9.2 rev/s at the position 1, which might be contributed to the immediate adjacency between position 1 and the cilium, where photonic energy density reaches maximal values due to reduced spatial attenuation.

1. **The calculated *ω* as a function of the number of OPWs.**

**
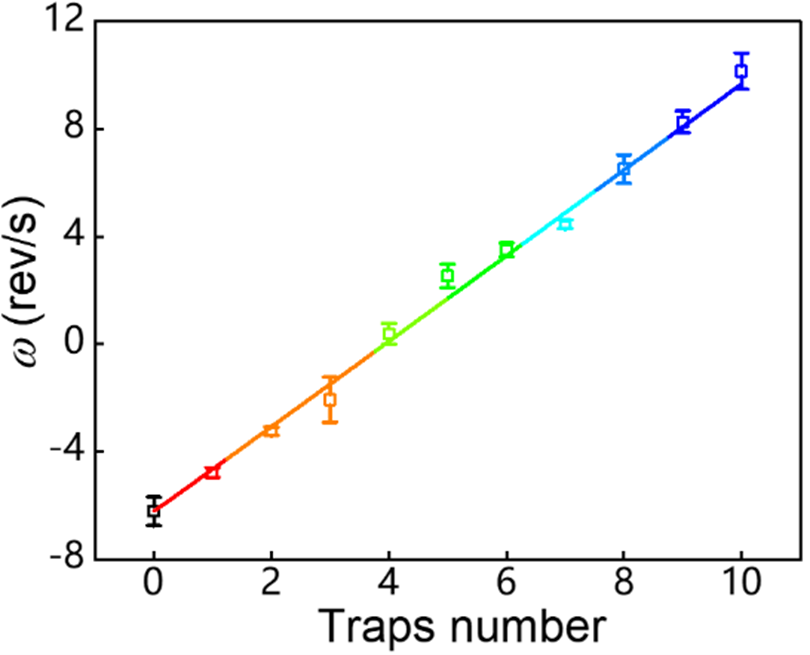
**

**Figure S12.** The calculated *ω* as a function of the number of OPWs. The rotational speed exhibited a linear increase with the number of OPWs, which should contribute to the enhanced stimulation by the cumulative photon absorption.

1. **Quantitative analysis of ciliary length, diameter and rotational speed in embryo LRO after incubation with methylcellulose.**

**
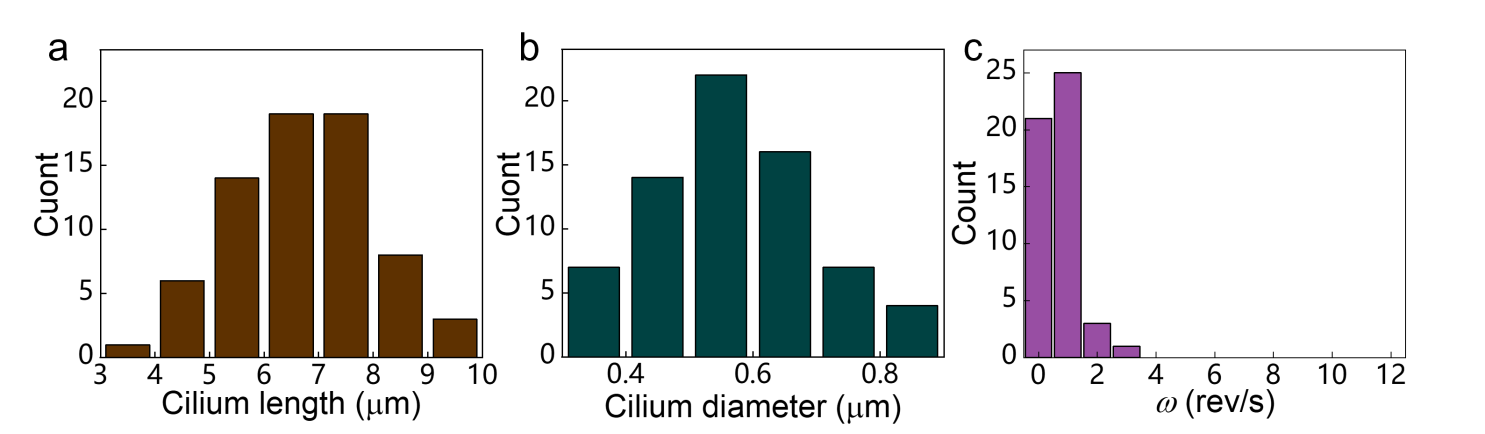
**

**Figure S13.** Quantitative analysis of ciliary length, diameter and rotational speed in embryo LRO after incubation with methylcellulose.

1. **The flow velocity of recirculating microfluid within the LRO.**


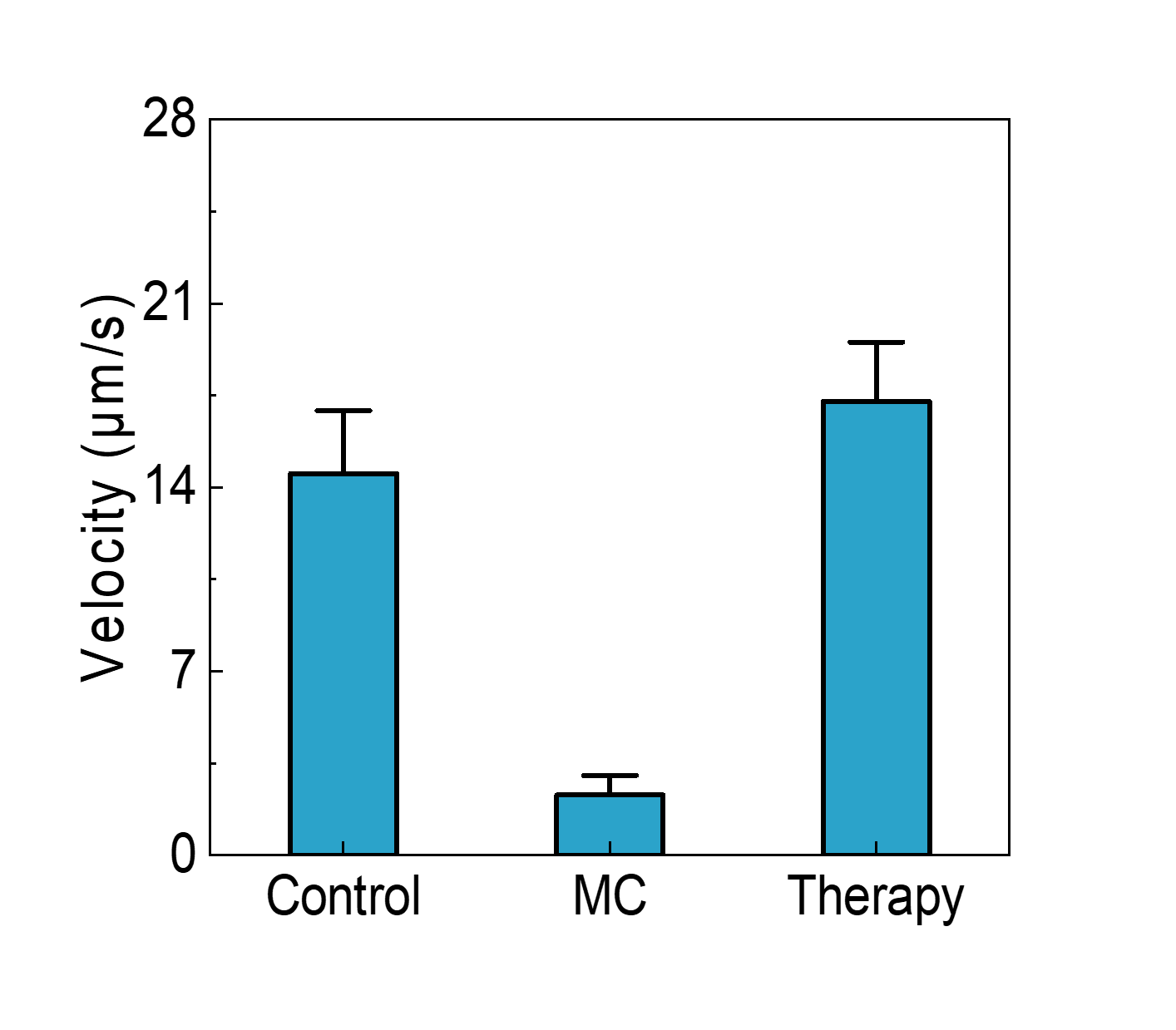


**Figure S14.** The flow velocity of recirculating microfluid within the LRO.

1. **Quantitative characterization of optically manipulated single cilium on cardiac looping directionality.**


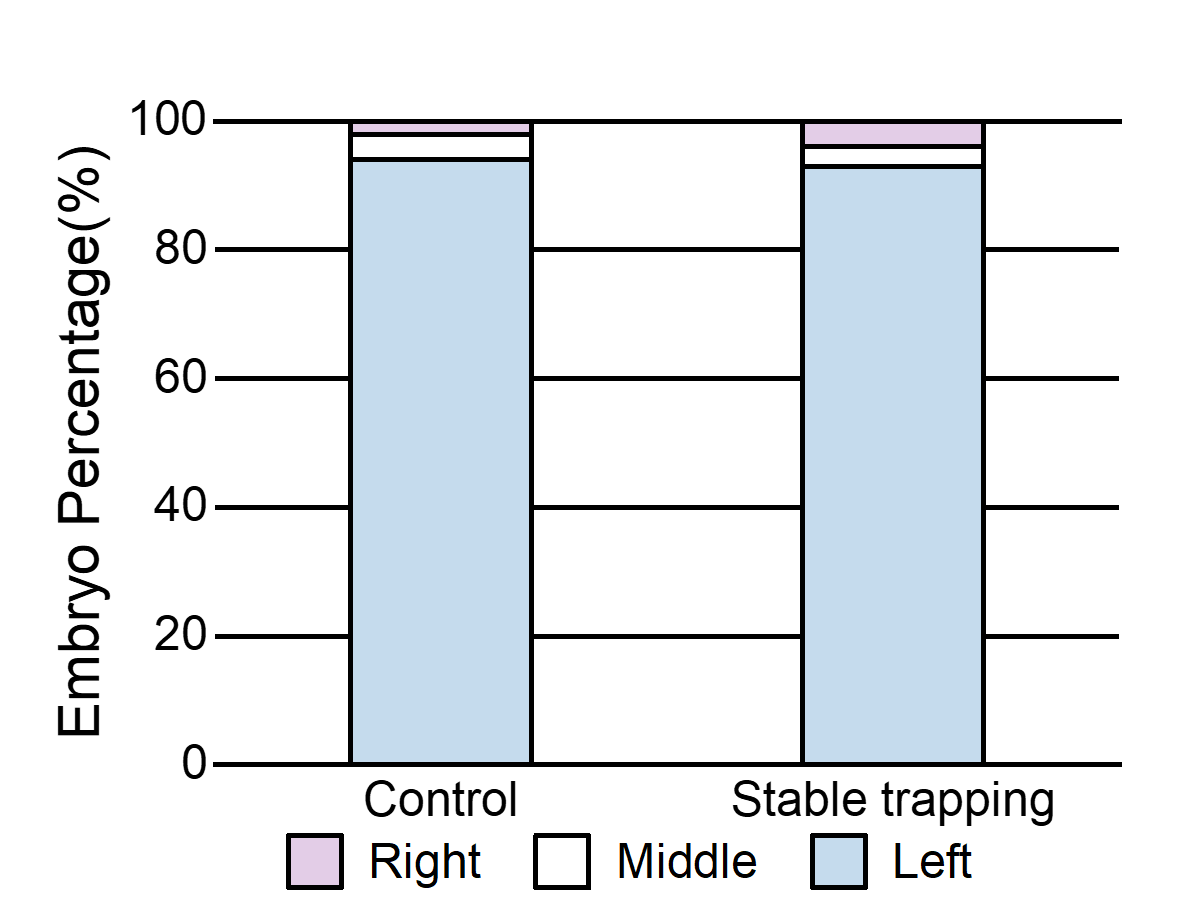


**Figure S15.** Quantitative characterization of optically manipulated single cilium on cardiac looping directionality. The OPWs were employed to stably trap single cilia within LRO, maintaining immobilization for >1 hour while neighboring motile cilia continued rotating. Post-manipulation embryonic development was longitudinally monitored, which demonstrates cardiac looping direction exhibited no significant deviation relative to the case of single-cilium manipulation.

1. **Optical manipulation of the cilia within the inner ear.**


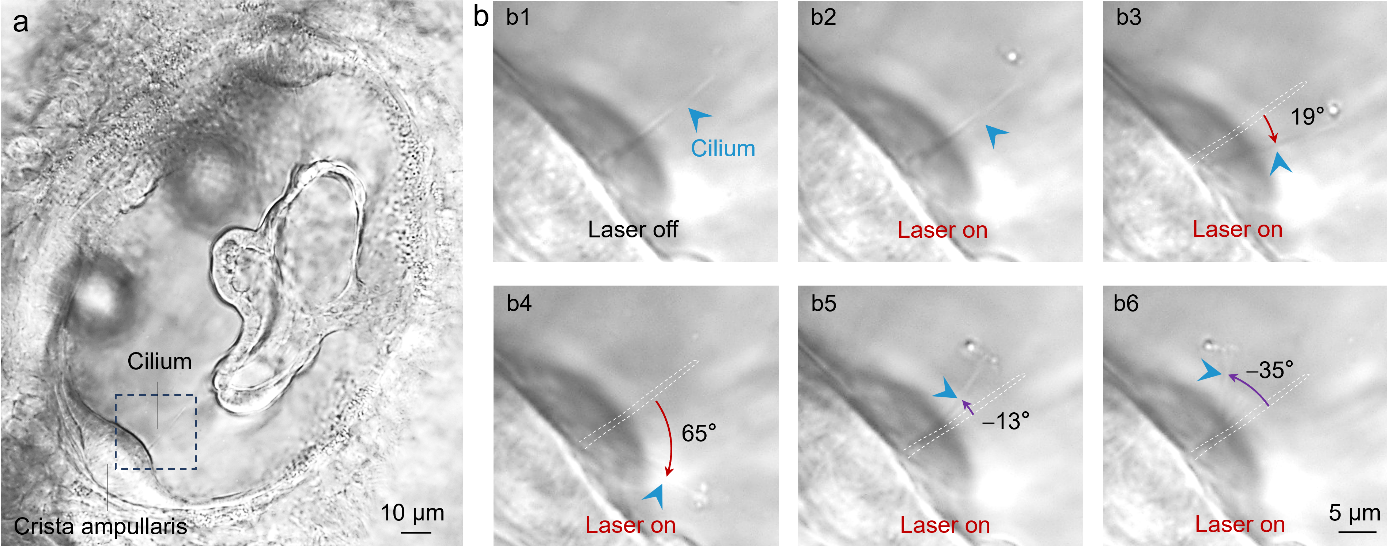


**Figure S16**. Optical manipulation of the cilia within the inner ear. (a) Representative optical micrograph of the zebrafish inner ear. (b) Bidirectionally optical deflection of cilia on the crista ampullaris.

1. **Biosafety characterization of the Opto-Bio-Hydrodynamic platform.**


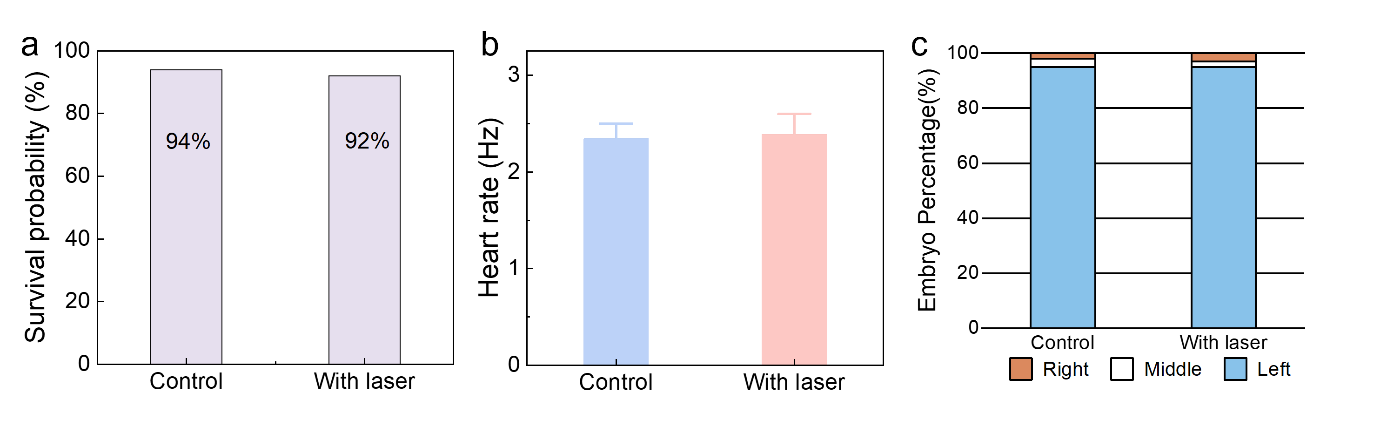


**Figure S17**. Comparative analysis of embryonic development including survival rate (a), heart rate (b) and spatial patterning of cardiac development (c) for the control group and optically manipulated group.
